# Acute elevated dietary fat alone is not sufficient to decrease AgRP projections in the paraventricular nucleus of the hypothalamus in mice

**DOI:** 10.1038/s41598-024-70870-0

**Published:** 2024-08-29

**Authors:** Selma Yagoub, Robert A. Chesters, Jonathan Ott, Jiajie Zhu, Lídia Cantacorps, Katrin Ritter, Rachel N. Lippert

**Affiliations:** 1 German Institute for Human Nutrition Potsdam-Rehbrücke, Nuthetal, Germany; 2 University of Potsdam, Potsdam, Germany; 3 German Center for Diabetes Research (DZD), Neuherberg, Germany; 4 NeuroCure Cluster of Excellence, Charité – Universitätsmedizin Berlin, Berlin, Germany

**Keywords:** AgRP, High fat diet, Axonal projections, Paraventricular nucleus of the hypothalamus, Melanocortin, Neural circuits, Obesity, Hypothalamus, Obesity

## Abstract

Within the brain, the connections between neurons are constantly changing in response to environmental stimuli. A prime environmental regulator of neuronal activity is diet, and previous work has highlighted changes in hypothalamic connections in response to diets high in dietary fat and elevated sucrose. We sought to determine if the change in hypothalamic neuronal connections was driven primarily by an elevation in dietary fat alone. Analysis was performed in both male and female animals. We measured Agouti-related peptide (AgRP) neuropeptide and Synaptophysin markers in the paraventricular nucleus of the hypothalamus (PVH) in response to an acute 48 h high fat diet challenge. Using two image analysis methods described in previous studies, an effect of a high fat diet on AgRP neuronal projections in the PVH of male or female mice was not identified. These results suggest that it may not be dietary fat alone that is responsible for the previously published alterations in hypothalamic connections. Future work should focus on deciphering the role of individual macronutrients on neuroanatomical and functional changes.

## Introduction

Feeding responses are predominantly regulated within the brain via Agouti-related peptide (AgRP) and proopiomelanocortin (POMC) neurons, two neural populations of the central melanocortin system. They are located mainly in the arcuate nucleus of the hypothalamus (ARC) and have opposite actions on regulating feeding. When activated, POMC neurons inhibit food intake whilst AgRP neurons increase it. These neurons also express receptors to metabolic hormones such as insulin, leptin and ghrelin, that allow them to continuously monitor the energy state of the animal and alter their activity to maintain energy homeostasis. In this context, periods of fasting, or prolonged food deprivation are known to decrease circulating levels of leptin and insulin. Fasting also increases the activity of arcuate AgRP neurons^1,2^ while refeeding behaviour after 24 h fasting is prevented if these AgRP neurons are ablated^3^. Furthermore, AgRP neuron activity changes in response to energetically dense foods. This change in activity may be dependent on the length of HFD exposure. For example, long term access to high fat diet (HFD) compromises AgRP sensitivity to hormonal signals such as ghrelin or leptin, contributing therefore to the disruption of energy balance and resulting in leptin resistance^4–6^. Moreover, acute 48 h exposure to HFD has been shown to cause a loss of AgRP neuron leptin sensitivity^7,8^. Conversely however, a recent electrophysiological study has shown an increase in AgRP neuronal activity upon an acute exposure to HFD for 2 days^9^.

The consequences of long term (greater than 8 weeks) HFD exposure on the function of the hypothalamic melanocortin system has been extensively studied, but predominantly only in male mice. Electrophysiology and fiber photometry studies have shown that AgRP/ Neuropeptide-Y (NPY) neurons become hyperexcitable after 8 weeks of HFD in male mice^7,10^. In females, the same time exposure also induces AgRP neuron hyperexcitability but to a lesser extent because of an elevated baseline firing rate compared to males ^9,10^. Moreover, in male mice, it has been shown that 8 weeks of HFD is sufficient to cause a dramatic, 60% decrease in AgRP axonal projections to the paraventricular nucleus of the hypothalamus (PVH), a nucleus where the action of AgRP release on their receptor, the melanocortin receptor 4 (MC4R) neurons is known to be the dominant driver of food intake^7^. Interestingly, and most surprisingly, short-term HFD exposure, as short as 48 h, has been reported to have an even greater effect on the reduction in AgRP neuronal projections, with an 80% decrease in projections observed in the PVH of male mice^7^. As in the long term HFD exposure, functional changes in AgRP neuronal activity have also been observed following 48 h of HFD, with a rapid change in calcium activity seen in response to the presentation of food^11–13^ as well as increased neuronal firing of AgRP neurons^7,14^. Additionally, 48 h HFD led to an up-regulation of suppressor of cytokine signaling-3 (SOCS3), an inflammatory and insulin signalling hallmark in AgRP but not in POMC neurons in the ARC^8^. Another study showed that 24 h intralipid treatment in the ARC revealed an up-regulation of TNF-α and an increase in the number of astrocytes mirroring neuroinflammation in that nucleus^15^. Furthermore, hypothalamic proteomics data collected from 72 h HFD-exposed male mice highlighted a change in protein spots involved in neuronal remodelling and synaptic plasticity indicating a structural adaptation of the hypothalamus^16^. Altogether, it seems that in male mice short term HFD exposure presents similar electrophysiological, molecular and structural signature changes in AgRP neurons as long term HFD exposure.

Despite the growing evidence pointing toward sex-specific metabolic and central adaptations (e.g. firing rate, neuronal connections) in response to a long term HFD exposure^17–23^, the effect of a short term HFD exposure in female mice is still poorly studied in comparison to males. Here, we explored the potential sex-specific adaptative response to an acute (48 h) HFD exposure on AgRP axonal projections within the hypothalamus in male and female mice. We specifically targeted dietary fat, and not elevated sucrose as commonly found in HFD, to ensure that the effects would be attributed to increased fat consumption alone. We assessed neuroanatomical changes using both the endogenous AgRP peptide as well as a targeted labeling of a synaptic protein. Image analyses utilized two pipelines designed based on previous literature. Our findings using both image analyses pipelines show no change of AgRP neuronal connections in the PVH after 48 h of HFD both in male as well as female mice. Thus, acute elevations in dietary fat alone are not sufficient to modify AgRP axonal architecture in the PVH.

## Results

### 48 h HFD exposure does not markedly change metabolic parameters in mice

**Fig. 1:**
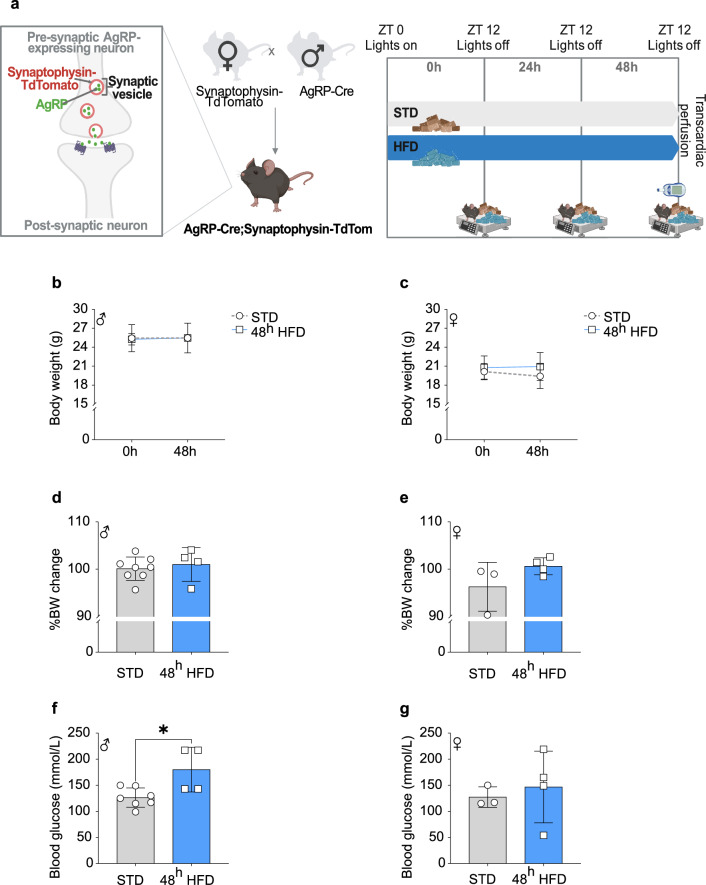
Metabolic parameters in response to 48 h access to HFD. (**a**) Mouse model and experimental time line schema (Created with BioRender.com). (**b**) Body weight at start (0 h) and end (48 h) of the experiment in male and (**c**) female mice. (**d**) Percentage of body weight change after 48 h compared to 0 h in STD and 48 h HFD male and (**e**) female mice. (**f**) Random fed blood glucose measurements at sacrifice in STD and 48 h HFD male and (**g**) female mice. All measurements were performed at starting Zeitgeber time 12 (ZT 12) or onset of the dark phase. STD: males n = 8; females n = 3, HFD: males n = 4; females n = 4. Statistical analysis performed using an unpaired *t*-test. *p-value < 0.05.

The effects of HFD on AgRP neurons are often attributed the increased dietary fat alone. However, the high fat diets used in previous studies are often confounded by the presence of increased dietary sucrose as well^7,10,24^. To overcome this and to specifically study the effects attributed to elevated dietary fat, we utilized a 60% HFD with similar sucrose to animals receiving only a standard diet (STD: 9% kCal from fat). The HFD-exposed group were given access to HFD at the onset of the dark phase (Zeitgeber time (ZT) 12) for 48 h until sacrifice (Fig. 1a). There were no significant changes in body weight after 48 h HFD exposure, in either male (Fig. 1b,d) or female animals (Fig. 1c,e). In males (Fig. 1f) but not in females (Fig. 1g), random fed detection of glycemia was significantly increased after 48 h of access to HFD (Males: 18.49 ± SD vs. 42.72 ± SD, *p* = 0.0161, Females: 19.66 ± SD vs. 68.70 ± SD, *p* = 0.6614).

### AgRP neuronal projections in the PVH are unchanged after 48 h HFD exposure

It has been shown that HFD exposure induces sex-specific responses particularly in the hypothalamus^17–19,22,23^. It has been reported that in male mice, 48 h of HFD access is sufficient to decrease 80% of AgRP axonal projections to the PVH^7^. However, no studies have investigated the effect in female mice. In order to compare the effect of a short-term HFD exposure on AgRP projections to the PVH in male and female mice, we used AgRP-IRES-Cre; Synaptophysin-TdTomato adult mice. This mouse model allowed us to assess the synaptic protein, Synaptophysin, specifically in AgRP neuronal projections through detection of TdTomato signal from the Synaptophysin-TdTomato fusion protein expression. Image analyses were performed in accordance with previously literature using both a maximum intensity projection method and sum of slices method^7,25,26^. In both analysis pipelines the first step to process the acquired images for analysis is to set a threshold for each image. This thresholding value utilizes an automatic detection of overall signal intensities across the image and uses an algorithm to apply a standardized selection of positively labeled signal to generate a binary image. For each image analysis method, we compared between STD and 48 h HFD groups the automatically generated thresholding values applied to AgRP and Synaptophysin-TdTomato images. AgRP and Synaptophysin-TdTomato thresholding values were not significantly different between STD and 48 h HFD groups (Supp. Figure 1, Supp. table 1).

**Fig. 2:**
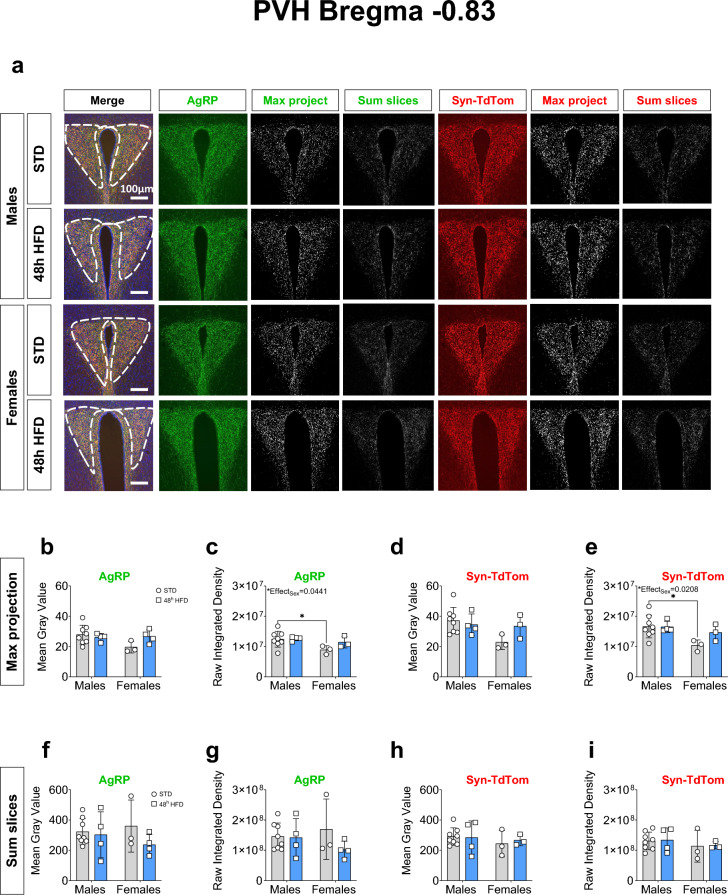
48 h HFD does not decrease AgRP connections in anterior PVH in adult male and female mice. (**a**) Representative images of AgRP, Synaptophysin-TdTomato and DAPI with the region of interest (ROI) representing the PVHant, AgRP (green), Synaptophysin-TdTomato (red) immunostaining images, the maximum intensity projection and the sum of the Z-stack slices in the PVHant (bregma -0.83) in male (STD: n = 8; HFD: n = 4) and female (STD: n = 3; HFD: n = 4) mice exposed to either HFD or STD for 48 h. (**b**–**e**) Comparative analysis between males and females of the thresholded maximum intensity projection images of AgRP and (**d, e**) Synaptophysin-TdTomato immunostaining represented as (**b**–**d**) Mean gray value and (**c**–**e**) Raw Integrated Density values. (**f**–**i**) Comparative analysis between males and females of the thresholded Z-stack images of AgRP and Synaptophysin-TdTomato immunostaining represented as (**d**–**h**) Mean gray value and (**g**–**i**) Raw Integrated Density. The ROI was drawn around the PVH in reference to the mouse brain atlas ^27^. Statistical analysis was performed using a Two-way ANOVA followed by Bonferroni post hoc test in c and e. *p < 0.05.

As the cellular identity and heterogeneity of the PVH dramatically differs across the anterior to posterior axis, we first, assessed AgRP and Synaptophysin-TdTomato in the PVH anterior (PVHant) (Fig. 2a)^27^. This region corresponds to the neuroendocrine compartment of the PVH, a region enriched in receptors for a number of neuropeptide hormones such as MC4R^28,29^. Using the maximum intensity projection method of analysis, the most common approach used in the literature, we did not detect any sex-specific difference in the mean gray value of AgRP and Synaptophysin-TdTomato labeling (Fig. 2b and d). However, analysis of the Raw Integrated Density of AgRP and Synaptophysin-TdTomato signals did highlight an overall effect of sex, with a significant difference between male and female animals on STD (Fig. 2c, e, Table 1). Contrary to the published literature, we did not detect any significant reduction in AgRP projections after 48 h of HFD exposure in the PVHant in neither males nor females, regardless of the analysis method used (Fig. 2 and Table 1).

**Table 1:** Two-way ANOVA statistical analysis of data presented in Fig. 2.

Panel	Source of variation	F (DFn, DFd)	*p*-value
Method 1: Maximum intensity projection			
(b) AgRP mean gray value	Interaction	"F (1, 15) = 3.131"	0.0971
	Sex	"F (1, 15) = 2.396"	0.1425
	Diet	"F (1, 15) = 1.134"	0.3037
(c) AgRP Raw Integrated Density	Interaction	"F (1, 14) = 1.277"	0.2774
	Sex	"F (1, 14) = 4.895"	0.0441*
	Diet	"F (1, 14) = 1.638"	0.2214
(d) Syn-TdTom mean gray value	Interaction	"F (1, 14) = 2.858"	0.1130
	Sex	"F (1, 14) = 3.674"	0.0759
	Diet	"F (1, 14) = 0.9927"	0.3360
(e) Syn-TdTom Raw Integrated Density	Interaction	"F (1, 14) = 2.000"	0.1791
	Sex	"F (1, 14) = 6.781"	0.0208*
	Diet	"F (1, 14) = 1.761"	0.2057
Method 2: Sum slices			
(f) AgRP mean gray value	Interaction	"F (1, 15) = 0.8681"	0.3662
	Sex	"F (1, 15) = 0.06371"	0.8041
	Diet	"F (1, 15) = 1.665"	0.2164
(g) AgRP Raw Integrated Density	Interaction	"F (1, 15) = 1.321"	0.2684
	Sex	"F (1, 15) = 0.08496"	0.7747
	Diet	"F (1, 15) = 1.673"	0.2154
(h) Syn-TdTom mean gray value	Interaction	"F (1, 14) = 0.1469"	0.7072
	Sex	"F (1, 14) = 0.7393"	0.4044
	Diet	"F (1, 14) = 0.04054"	0.8433
(i) Syn-TdTom Raw Integrated Density	Interaction	"F (1, 14) = 4.327e-005"	0.9948
	Sex	"F (1, 14) = 0.9702"	0.3413
	Diet	"F (1, 14) = 0.02912"	0.8669

* = p < 0.05

**Fig. 3:**
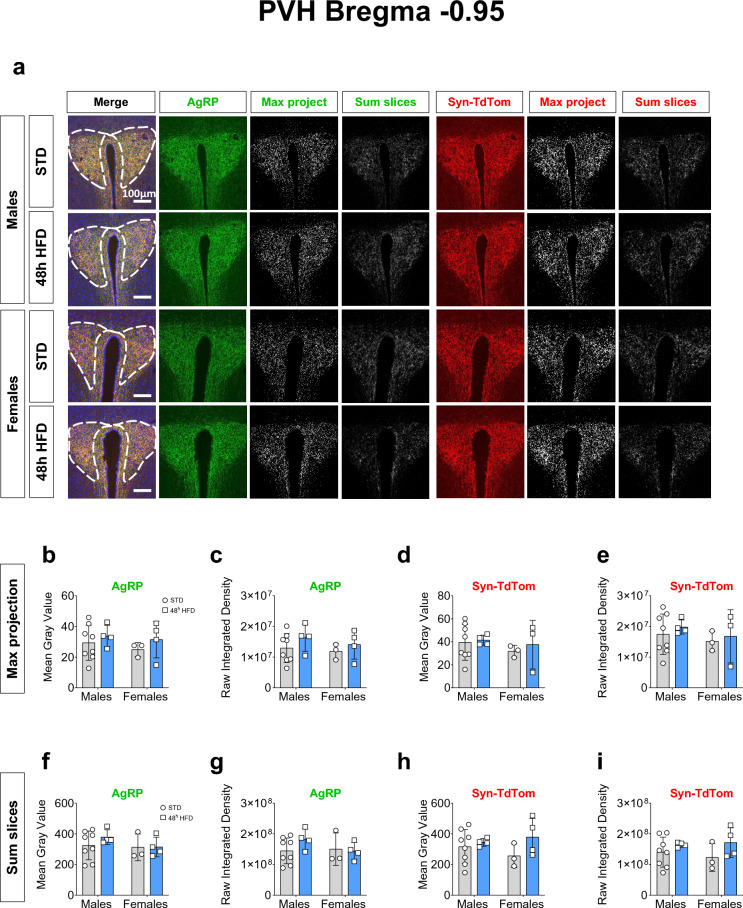
48 h HFD does not decrease AgRP connections in mid PVH in adult male and female mice. (**a**) Representative images of AgRP, Synaptophysin-TdTomato and DAPI with the region of interest representing the PVHmid, AgRP (green), Synaptophysin-TdTomato (red) immunostaining images, the maximum intensity projection and the sum of the Z-stack slices in the PVHmid (bregma − 0.95) in male (STD: n = 8; HFD: n = 4) and female (STD: n = 3; HFD: n = 4) mice exposed to either HFD or STD for 48 h. (**b**–**e**) Comparative analysis between males and females of the thresholded maximum intensity projection images of AgRP and (**d**–**e**) Synaptophysin-TdTomato immunostaining represented as (**b**–**d**) Mean gray value and (**c**–(**e**) Raw Integrated Density values. (**f**–**i**) Comparative analysis between males and females of the thresholded Z-stack images of AgRP and Synaptophysin-TdTomato immunostaining represented as (**d**–**h**) Mean gray value and (**g**–**i**) Raw Integrated Density. The ROI was drawn around the PVH in reference to^27^. Statistical analysis was performed using a Two-way ANOVA.

Next, we assessed AgRP and Synaptophysin projections in the midpoint of the PVH (PVHmid) (Fig. 3a)^27^. By applying the maximum intensity projection method of image analysis, the labeling of AgRP and Synaptophysin-TdTomato remained unchanged after 48 h access to HFD with no significant difference of AgRP projections between males and females in the mean gray value (Fig. 3b,d, Table 2b and d) and Raw Integrated Density (Fig. 3c,e, Table 2 c and e). The sum slices analysis method also did not detect any difference in AgRP and Synaptophysin labeling (Fig. 3f–i) and no effect of the diet on the projections (Table 2f–i). In sum, our results show no effect of 48 h HFD exposure on AgRP projections in the PVHmid.

**Table 2:** Two-way ANOVA Statistical analysis of data presented in Fig. 3.

Panel	Source of variation	F (DFn, DFd)	*p*-value
Method 1: Maximum intensity projection			
(b) AgRP mean gray value	Interaction	"F (1, 15) = 0.04563"	0.8337
	Sex	"F (1, 15) = 0.4600"	0.5080
	Diet	"F (1, 15) = 1.184"	0.2937
(c) AgRP Raw Integrated Density	Interaction	"F (1, 15) = 0.07372"	0.7897
	Sex	"F (1, 15) = 0.5952"	0.4524
	Diet	"F (1, 15) = 1.645"	0.2191
(d) Syn-TdTom mean gray value	Interaction	"F (1, 14) = 0.07452"	0.7889
	Sex	"F (1, 14) = 0.6688"	0.4272
	Diet	"F (1, 14) = 0.2914"	0.5978
(e) Syn-TdTom Raw Integrated Density	Interaction	"F (1, 14) = 0.01304"	0.9107
	Sex	"F (1, 14) = 0.7729"	0.3942
	Diet	"F (1, 14) = 0.4367"	0.5195
Method 2: Sum slices			
(f) AgRP mean gray value	Interaction	"F (1, 15) = 0.4469"	0.5139
	Sex	"F (1, 15) = 0.9467"	0.3460
	Diet	"F (1, 15) = 0.5240"	0.4803
(g) AgRP Raw Integrated Density	Interaction	"F (1, 15) = 1.286"	0.2745
	Sex	"F (1, 15) = 0.7140"	0.4114
	Diet	"F (1, 15) = 0.6582"	0.4299
(h) Syn-TdTom mean gray value	Interaction	"F (1, 14) = 0.2569"	0.6201
	Sex	"F (1, 14) = 0.5981"	0.4522
	Diet	"F (1, 14) = 1.224"	0.2871
(i) Syn-TdTom Raw Integrated Density	Interaction	"F (1, 14) = 0.01766"	0.8962
	Sex	"F (1, 14) = 0.4794"	0.5000
	Diet	"F (1, 14) = 1.628"	0.2227

#### 48 hour HFD exposure does not change postsynaptic markers

**Fig. 4:**
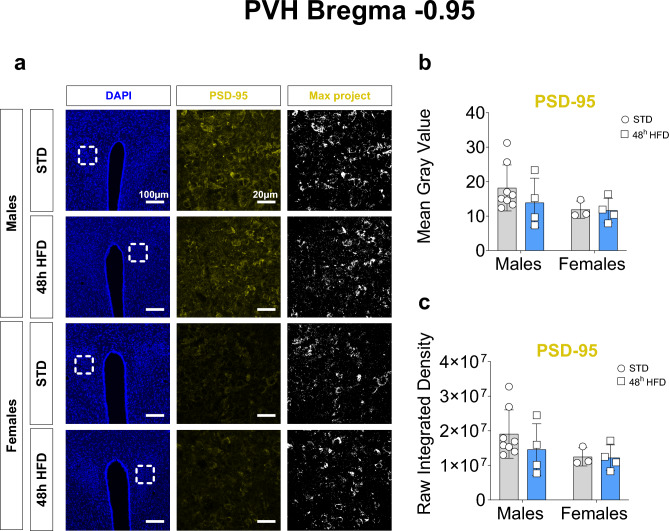
48 h HFD does not affect post-synaptic connections in the mid PVH in adult male and female mice. (**a**) Representative images of DAPI (blue) at 20× magnification showing the PVHmid, PSD-95 signal (yellow) and maximum intensity projection at 63 × magnification images of male (STD: n = 8; HFD: n = 4) and female (STD: n = 3; HFD: n = 4) mice exposed to either HFD or STD for 48 h. The square region represents the location of the image taken at 63 × magnification. (**b**, **c**) Comparative analysis between males and females of the thresholded maximum intensity projection images of Syn-TdTom as (**b**) Mean gray value and (**c**) Raw Integrated Density values. Statistical analysis was performed using a Two-way ANOVA.

While AgRP and synaptophysin are unchanged by HFD exposure, we aimed to test how the post-synapse may respond to the dietary manipulation. To do this, the postsynaptic protein, PSD-95 was measured in the PVHmid sections to assess changes to excitatory postsynapses. We first confirmed no change to synaptophysin-tdTomato signal at higher magnifications (Supplemental Fig. 2). For PSD-95, the response to 48 h of HFD exposure did not result in any significant effect of the diet or sex difference for both mean gray value and raw integrated density signal (Fig. 4 and Table 3).

**Table 3:** Two-way ANOVA Statistical analysis of data presented in Fig. 4.

PVH Bregma − 0.95			
Panel	Source of variation	F (DFn, DFd)	*p*-value
(b) PSD-95 mean gray value	Interaction	"F (1, 15) = 0.4954"	0.4923
	Sex	"F (1, 15) = 2.227"	0.1564
	Diet	"F (1, 15) = 0.6279"	0.4405
(c) PSD-95 Raw Integrated Density	Interaction	"F (1, 15) = 0.4954"	0.4923
	Sex	"F (1, 15) = 2.227"	0.1564
	Diet	"F (1, 15) = 0.6279"	0.4405

## Discussion

The aim of our study was to investigate sex-specific metabolic and neuroanatomical changes in AgRP projections to the PVH in response to acute 48 h HFD exposure. We specifically analysed AgRP neuronal projections to the anterior and middle PVH (PVHant and PVHmid), as this is the neuroendocrine compartment of the PVH, and is known to express the MC4R; the main target of AgRP release in this region^29–31^. In accordance with the literature, we did not see an effect of acute 48 h HFD exposure on body weight^16,32^. However, blood glucose levels were significantly increased in male mice. Since blood glucose was measured in *ad-libitum* fed mice, this minor, but significant difference may be due to the consumption of HFD shortly before sacrifice: as the animals were sacrificed after onset of the dark phase (ZT 12), when mice usually consume a large majority of their daily food intake.

Next, we investigated the effects of 48 h HFD on AgRP projections using an AgRP-Cre; Synaptophysin-TdTomato mouse model specifically labeling these projections. To analyse images of AgRP and Synaptophysin-TdTomato, we applied two methods of neuronal projection analysis published in the literature: the maximum intensity projection and sum slices (described in material and methods section)^7,25,26^. In the PVHant and using the maximum intensity method, our analysis of the Raw Integrated Density of AgRP and Synaptophysin-TdTomato revealed a significant sex difference but ultimately no significant effect of the 48 h HFD exposure within each sex. This sex effect seems to be driven primarily by a difference within the STD-exposed groups. In the PVHmid, no significant effect of sex nor diet were observed.

Methodological factors that can potentially influence the results are: method of thresholding (manual versus automated), what defines the Region of Interest (ROI) drawn on the image, and/or if the same approach to slice analysis is used (single comparable slice versus average of multiple slices). In our study, we standardized the coronal brain slice selected, the ROI used, and selected an automated unbiased thresholding algorithm (ImageJ—Moments). We also quality checked all thresholded binarized images to ensure the accurate inclusion of all positively-labeled pixels. Furthermore, we checked that automatic thresholding values were not significantly different from each other across both sexes and diet groups. While using the maximum intensity projection and sum of slices for confocal image analyses, we observed a higher accuracy between the binarized image and the original image using the maximum intensity projection. It was observed that with the sum of slices method, there was an over estimation of the positive signal, including signal from the background and not specifically the positively labeled neuronal projections. Therefore, we would recommend applying the maximum intensity projection method for analysing neuronal projections. Regarding the region of analysis representing the PVH, we differentiated the anterior and mid PVH to localize and target the effect of HFD on AgRP projections due to the known intra-PVH complexity and likely differing functions of subregions of the PVH^24,29,30^.

To determine if potential effects could be mediated by changes to the post-synapse, we measured labeling of PSD-95, a marker of excitatory synapses. MC4R is known to accumulate in close proximity to PSD-95 in excitatory synapses^33^ which is thought to be driving satiety signaling in this circuit despite the relatively low amount of glutamatergic versus GABAergic input from the arcuate nucleus^34,35^. Here we also found no differences between the control and acute HFD exposed groups. This supports that postsynaptic alterations are also not affected by elevated fat in the diet in the acute sense.

Our results demonstrate that acute exposure to elevated amounts of dietary fat, alone, does not alter AgRP axonal projections to the PVH. This holds true for both males and females. An interesting point that is still open for discussion is the contribution of fat alone, or fat in the presence of elevated sugar content as well as the cause for synaptic change. The primary study showing a dramatic decrease in AgRP projections to the PVH used a diet high in fat (45%) but also in sugar (17% sucrose)^7^. It was previously shown that AgRP neurons adjust their response to acute high fat and high sucrose, through electrophysiological and calcium dynamics or even AgRP mRNA levels^7,10,13,36^. Moreover, a recent publication highlighted electrophysiological changes upon acute dietary exposures appear to be predominantly due to the excess dietary sucrose^14,37^, underscoring that there are unique responses of AgRP neurons to macronutrient content in the diet. While our primary focus was to decipher the contribution of elevated fat to the diet, and thus we only utilized a diet high in fat content, it is still necessary to determine if the elevated sucrose paired with high fat is potentially driving changes to AgRP synapses which were previously reported. This evidence raises the question as to the contribution of individual macronutrient contents, separately and/or in combination, to induce synaptic changes within the hypothalamus and the brain generally. Therefore, understanding the role of individual macronutrients acquired through the diet, on aspects of synaptic physiology may give insight into the acute adaptability of neuronal circuits in adjusting to nutritional changes.

## Materials and methods

### Animals

AgRP-IRES-Cre male mice (strain AgRPtm1(cre)Lowl/J, Jackson laboratories Stock number:012899) were crossed with ROSA-CAG-LSL-Synaptophysin-tdTomato-WPRE^(Ai34D flox/flox)^ female mice (Strain B6;129S-Gt (RO-SA)26Sortm34.1 (CAG-Syp/tdTomato)Hze/J, Jackson Laboratories, Stock number: 012570) to obtain AgRP-IRES-Cre;Syn-TdTomato^flox/wt^ mice used in our experiments. Animals were group housed (2–3 per cage) with a 12-h light/dark cycle at 22 ± 2 °C and 50–70% humidity with ad libitum access to water and control STD (Ssniff # V1534-300; kCals metabolizable energy: 67% carbohydrates; 24% proteins and 9% fat; with 5.3% of total weight coming from crude sugar) unless otherwise stated. Mice were randomly assigned to either STD control or HFD for 48 h (HFD introduced at start of the dark phase) (Ssniff #EF acc. D12492 (I) mod; kCals metabolizable energy: 22% carbohydrates, 24% protein and 60% fat; with 9.4% of total weight coming from crude sugar). All animal experiments and procedures were approved by the animal welfare committee (Landesamt für Arbeitsschutz, Verbraucherschutz und Gesundheit; animal ethics application number 2347-36-2021). Experiments were conducted in accordance with the ARRIVE guidelines and the European Directive 2010/63/EU.

### Metabolic measurements

All measurements were performed during the dark phase at ZT 12. Body weight and food intake were measured at onset of the dark cycle on the first day of the experiment, 24 h and 48 h later before the sacrifice. Percentage body weight change was calculated as: body weight (g) at 48 h × 100/body weight (g) at start of the experiment. Glycemia was assessed at sacrifice by blood collection directly from the right atrium and measured with a glucometer (Contour Care, Ascensia).

### Immunostaining

Mice were deeply anesthetised with an intra-peritoneal injection of Pentobarbital (400 mg/kg) and sacrificed by transcardial perfusion with, first, 60 mL of ice cold 1X phosphate-buffered saline (PBS), then 4% paraformaldehyde (PFA, pH 9.5, 3.8% Borate) after onset of the dark phase ZT 12–15. Brains were post fixed in 4% PFA + Borax for 4 h then switched to a 20% sucrose solution overnight before being stored at − 80 °C until usage. Fixed brains were sliced coronally using a freezing sledge microtome (Slide 4004 M, pfm medical, Cat. 400,410) to obtain 30 μm frozen brain slices that were stored in anti-freeze solution (10% 10X PBS, 20% Ethyl glycol and 20% Glycerol) at − 20 °C until further use. The day of the staining, slices were washed 3 × 10 min times with 0.02 M KPBS, 5 min in 0.3% glycine in PBS and 10 min 0.03% SDS in PBS followed by a 1 h incubation in blocking and permeabilization buffer containing 3% donkey serum in 0.25% Triton-X in PBS. Slices were then incubated with primary antibodies Rabbit anti-AgRP (Phoenix; Cat# H-003-57; dilution 1:4,000), Goat anti-TdTomato (SICGEN, Cat# AB8181-200; dilution 1:4,000) and Rabbit anti-PSD-95 (ProteinTech; Cat# 20,665-1-AP; dilution 1:100) mixed in SignalStain® Antibody Diluent (CellsSignal; Cat# 8112) for 48 h at 4 °C plus 2 h at room temperature. After washing with 0.02 M KPBS (1 × 5 min and 3 × 10 min), slices were incubated with the secondary antibodies Donkey anti-Rabbit (1:500) Alexa Fluor 488-conjugated and Donkey anti-Goat (1:500) Alexa Fluor 546-conjugated diluted in 0.25% Triton-X in 1 × KPBS for 1 h at room temperature then washed with 0.02 M KPBS (1 × 5 min and 3 × 10 min). Brain slices were mounted on Superfrost Plus microscope slides and cover slipped with VECTASHIELD Antifade Mounting Medium with DAPI (Biozol; Cat# VEC-H-1200). Images of the PVHant (distance from Bregma -0.83 mm) and PVHmid (distance from Bregma -0.95 mm), selected based on the mouse brain atlas^27^, were collected using a confocal microscope (Multiphoton Laser Scanning Microscope LSM 780, Zeiss) and Zen (Zeiss) software. 3D Z-stack (z = 17 slices/image, interval = 1.5 µm) images were acquired. Two mice were excluded from further image processing and analysis due to obvious germline deletion of the transgene STOP cassette resulting in ubiquitous expression of Synaptophysin-TdTomato protein. For the PSD-95 imaging, a single 63X image in the PVHmid was collected for higher resolution imaging of the postsynaptic protein. Power/gain was determined by selecting 3 random slices from 3 different samples matched for the same distance from bregma and measuring the gain/power assigned for imaging of the samples. Once done, the average gain/power was calculated and this value was then assigned for use in the collection of all sample images. This allowed for an unbiased assignment of gain/power to the image analysis.

### Image processing and analysis

**Fig. 5:**
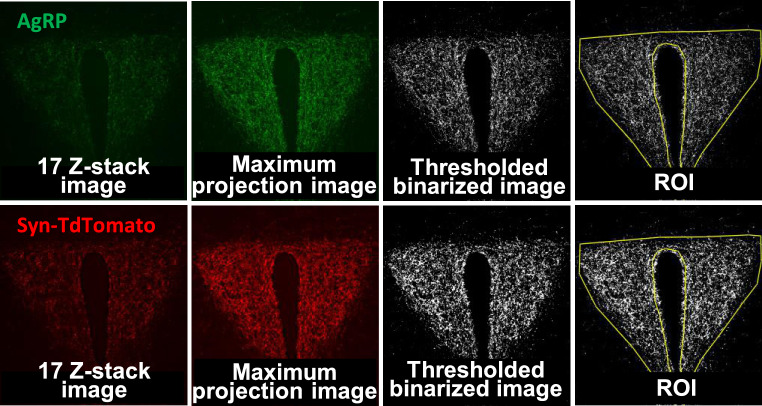
Workflow of image analysis using the ‘maximum projection’ analysis (left to right).

**Fig. 6:**
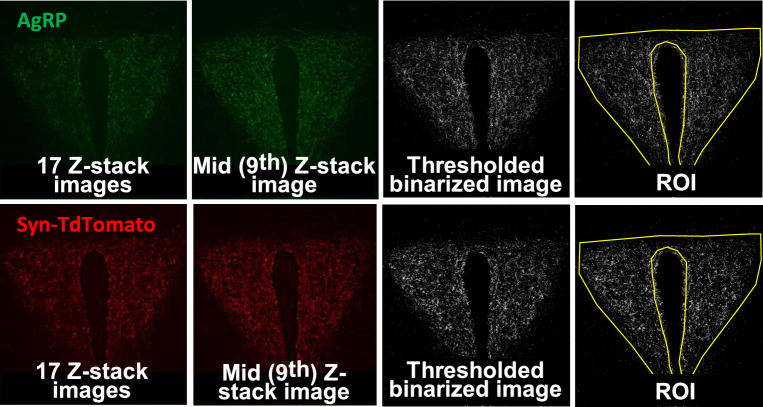
Workflow of image analysis using the ‘Sum slices’ analysis (left to right).

Images were processed and analyzed with a custom-written ImageJ macro and performed by an experimenter blinded to the experimental conditions. First, a region of interest (ROI) defining the outlines of the PVH was generated (Figs. 2 and 3a). This ROI was then used in the following two methods:

1. *Maximum intensity projection*: For each channel, Z-stack images were converted to a 2D image using a maximum intensity projection, and saved opened as individual files. A threshold value was automatically applied to these images using the “Moments” algorithm and a binary image created. The ROI was then overlayed on to the binary image. The workflow is represented in Fig. 5.
2. *Sum slices*: For each channel, a sum-slice intensity projection was created: this creates a 2D image by adding together the intensity of each pixel of each slice of the Z-stack at every x–y pixel coordinate in the 2D image space. The middle slice (number 9 out of 17) of the Z-stack was used as the reference slice and the sum-slice image, thresholded using “Moments” algorithm same this was applied to all 17 slices of the Z-stack image and a binary 2D image was saved. The resulting 32 bit image was analyzed after selecting the same ROI used for the maximum intensity projection method. The workflow is represented in Fig. 6.

For both methods, the size of the area, Raw Integrated Density (= sum of the pixels), and mean gray value (= sum of the gray values of all pixels divided by the number of pixels) were measured. Threshold values of the PVH ROI, from both analysis methods, were compared for differences across sexes and diet. All ImageJ scripts are available upon request. All ROIs and confocal images used for the maximum intensity projection and sum slices analyses can be shared upon request.

### Statistical analysis

All data are represented as mean + /− standard deviation (SD) with each single data point representing an individual mouse. GraphPad Prism 9 was used for all statistical analysis and graphs. An unpaired two-tailed *t*-test was conducted to analyse the body weight, the percentage change of body weight and blood glucose levels. AgRP projections were analysed with a two-way ANOVA to assess the sex, diet and Interaction of sex and diet. When appropriate, the two-way ANOVA was followed by Bonferroni post-hoc comparison. Threshold values were analyzed using a Mann Whitney comparison. Significant differences were considered when *p*-value < 0.05.

### Supplementary Information

Supplementary Information.

## Data Availability

All data will be made available upon reasonable request to the corresponding author.
