# Supplementary material for: Acute elevated dietary fat alone is not sufficient to decrease AgRP projections in the paraventricular nucleus of the hypothalamus in mice
Source: Sci Rep. 2024 Aug 29;14:20043. doi: 10.1038/s41598-024-70870-0 (PMC11362280; doi:10.1038/s41598-024-70870-0)
Supplement: Supplementary file 1 — Supplementary Information. [file 41598_2024_70870_MOESM1_ESM.pdf]

Supplementary Information

Title: Acute elevated dietary fat alone is not sufficient to decrease AgRP projections in the paraventricular nucleus of the hypothalamus in mice.

Selma Yagoub [1,2], Robert A. Chesters [1,4], Jonathan Ott[1], Jiajie Zhu [1,2,4], Lídia Cantacorps [1,3], Katrin Ritter [1], Rachel N. Lippert [1,3,4, *].

[1]German Institute for Human Nutrition Potsdam-Rehbrücke.

[2]University of Potsdam, Germany.

[3]German Centre for Diabetes Research (DZD), Neuherberg, Germany.

[4]NeuroCure Cluster of Excellence, Charité – Universitätsmedizin Berlin, Germany.

*Correspondence should be addressed to Rachel N. Lippert (Rachel.lippert@dife.de)

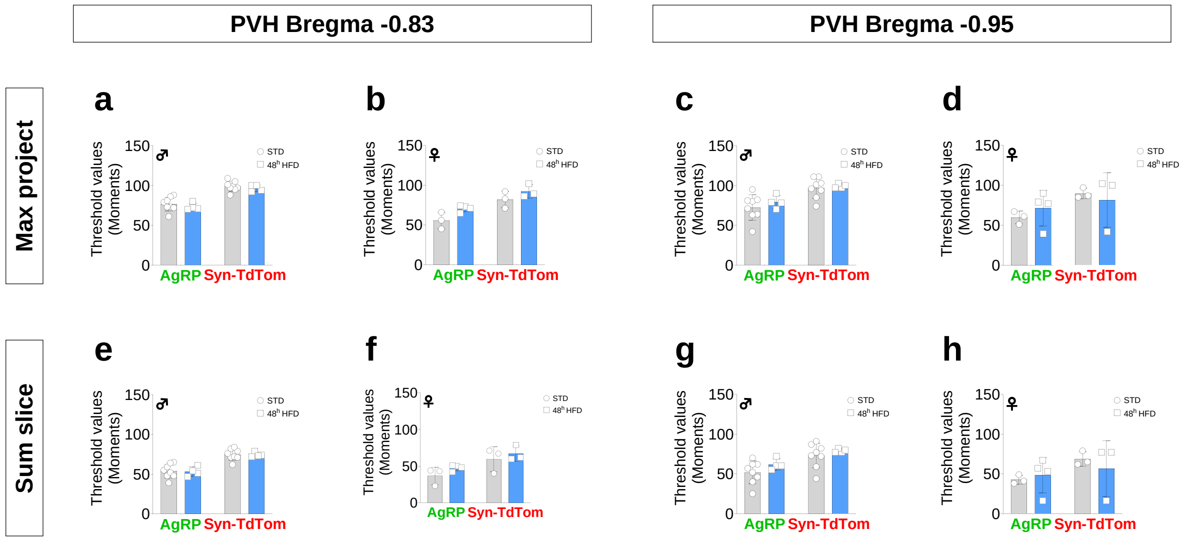

Supplementary Figure 1. Comparison of automated threshold detection values for PVH confocal images using ImageJ "Moments" Max Project or Sum slices pipelines. a) Automated ImageJ "Moments" thresholding values for AgRP and synaptophysin-TdTomato channels in the PVHant bregma -0.83 using Max Project analysis in males and **b)** females. **c)** Automated ImageJ "Moments" thresholding values for AgRP and synaptophysin-TdTomato channels in the PVHant bregma -0.95 using Max Project analysis in males and **d)** females. **e)** Automated ImageJ "Moments" thresholding values for AgRP and synaptophysin-TdTomato channels in the PVHant bregma -0.83 using Sum slices analysis in males and **f)** females. **g)** Automated ImageJ "Moments" thresholding values for AgRP and synaptophysin-TdTomato channels in the PVHant bregma -0.95 using Sum slices analysis in males and **h)** females. STD (males: n=8; Females n=4) and 48h HFD (males: n=3; Females n=3). Statistical analysis performed using a Mann-Whitney test separately for Max Project and Sum slices analysis.

Supplementary table.1. Mann-Whitney test statistical analysis of data presented in Supplementary Figure 1.

PVH Bregma -0.83			
**Panel**	**Analysis method**	**Source of comparison**	***p*-value**
a) Males: AgRP thresholded values<br>Males: Syn-TdTom	Max projection	STD vs 48h HFD	P= 0.2343<br>P= 0.5354
b) Females: AgRP thresholded values<br>Females: Syn-TdTom	Max projection	STD vs 48h HFD	P= 0.1143<br>P= 0.2286
e) Males: AgRP thresholded values<br>Males: Syn-TdTom	Sum slices	STD vs 48h HFD	P= 0.7758<br>P= 0.9152
f) Females: AgRP thresholded values<br>Females: Syn-TdTom	Sum slices	STD vs 48h HFD	P= 0.2286<br>P= >0.9999
**PVH Bregma -0.95**			
c) Males: AgRP thresholded values<br>Males: Syn-TdTom	Max projection	STD vs 48h HFD	P= 0.8081<br>P= 0.8384
d) Females: AgRP thresholded values<br>Females: Syn-TdTom	Max projection	STD vs 48h HFD	P= 0.4000<br>P= 0.7000
g) Males: AgRP thresholded values<br>Males: Syn-TdTom	Sum slices	STD vs 48h HFD	P= 0.3495<br>P= 0.6040
h) Females: AgRP thresholded values<br>Females: Syn-TdTom	Sum slices	STD vs 48h HFD	P= 0.4000<br>P= 0.7714

PVH Bregma -0.95

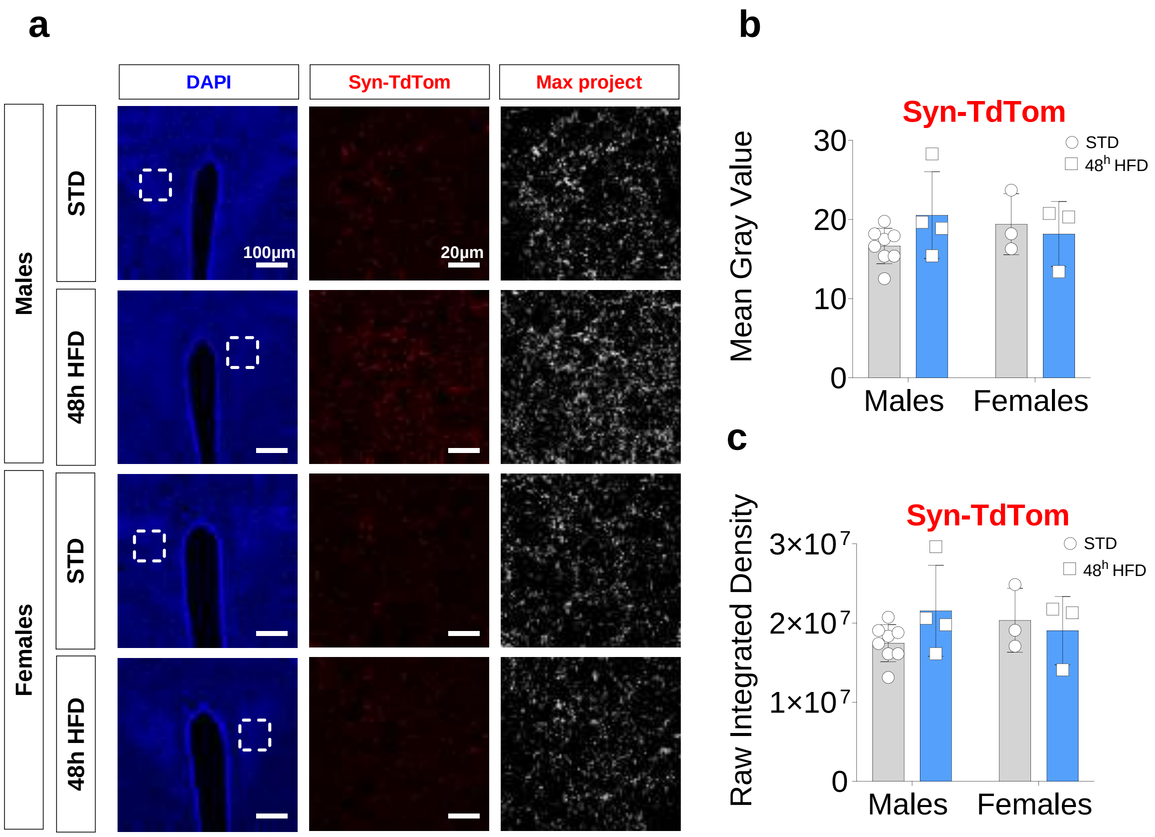

Supplementary Figure 2. 48h HFD does not affect pre-synaptic connections in the PVHmid in adult male and female mice. a) Representative images of a 20x confocal image of DAPI (blue) showing the PVHmid and the endogenous Syn-TdTom signal (red) of male (STD: n=8; HFD: n=4) and female (STD: n=3; HFD: n=4) mice exposed to either HFD or STD for 48h. The square region represents the location of the image taken at 63x magnification. **b-c)** Comparative analysis between males and females of the thresholded maximum intensity projection images of Syn-TdTom as **b)** Mean gray value and **c)** Raw Integrated Density values. Statistical analysis was performed using a Two-way ANOVA.

Supplementary table.2. Mann-Whitney test statistical analysis of data presented in Supplementary Figure 2.

PVH Bregma -0.95			
**Panel**	**Analysis method**	**Source of comparison**	***p*-value**
b) Syn-TdTom mean gray value	Interaction<br>Sex<br>Diet	"F (1, 14) = 1.876"<br>"F (1, 14) = 0.009285"<br>"F (1, 14) = 0.5043"	0.1923<br>0.9246<br>0.4893
c) Syn-TdTom Raw Integrated Density	Interaction<br>Sex<br>Diet	"F (1, 14) = 1.876"<br>"F (1, 14) = 0.009278"<br>"F (1, 14) = 0.5044"	0.1923<br>0.9246<br>0.4892
